# PBX2-Mediated circTLK1 Activates JAK/STAT Signaling to Promote Gliomagenesis via miR-452-5p/SSR1 Axis

**DOI:** 10.3389/fgene.2021.698831

**Published:** 2021-10-15

**Authors:** Jing Li, Zongren Zhao, Xiang Wang, Qiong Ma, Huanhuan Ji, Yan Wang, Rutong Yu

**Affiliations:** ^1^Institute of Nervous System Diseases, Xuzhou Medical University, Xuzhou, China; ^2^Department of Neurosurgery, Second People’s Hospital of Huai’an City, Huai’an Hospital Affiliated to Xuzhou Medical University, Huai’an, China; ^3^Department of Rehabilitation, The Affiliated Xuzhou Rehabilitation Hospital of Xuzhou Medical University, Xuzhou, China; ^4^Jiangsu College of Nursing, Huai’an, China

**Keywords:** circular RNA, circTLK1, miR-452-5p, SSR1, JAK/STAT signaling, glioma, PBX2

## Abstract

Glioma is considered one of the most lethal brain tumors, as the aggressive blood vessel formation leads to high morbidity and mortality rates. However, the mechanisms underlying the initiation and progression of glioma remain unclear. Here, we aimed to reveal the role of circTLK1 in glioma development. Our results revealed that circTLK1 is highly expressed in glioma tumor tissues and glioma cell lines. We then conducted a series of experiments that showed that circTLK1 was involved in the progression of gliomas. Mechanistically, investigation of the factors downstream of circTLK1 revealed that circTLK1 activated JAK/STAT signaling in glioma cells. Furthermore, AGO2-RIP, RNA-pull down, and luciferase reporter gene assays led to the identification of the novel circTLK1/miR-452-5p/SSR1 axis. Moreover, we investigated the upstream regulator of circTLK1 and found that circTLK1 expression in glioma cells could be regulated by the transcriptional factor PBX2. Taken together, our findings show that circTLK1 mediated by PBX2 activates JAK/STAT signaling to promote glioma progression through the miR-452-5p/SSR1 pathway. These results provide new insights into glioma diagnosis and therapy.

## Introduction

Glioma is one of the most common subtypes of malignant brain cancers and accounts for approximately 27% of central nervous system tumors (Louis et al., 2016). It is characterized by aggressive blood vessel formation, which leads to high morbidity and mortality (Van Meir et al., 2010; Khasraw et al., 2014). Despite clinical treatment of glioma, such as via surgery, combined chemotherapy, and radiotherapy, which have considerably improved in recent years, outcomes for patients remain unfavorable (Mangiola et al., 2010; Chen and Xu, 2016). Therefore, uncovering the mechanism underlying development of glioma is highly needed.

Circular RNAs (circRNAs) are 200–2,000 bp endogenous RNAs, which are transcribed by RNA polymerase II (Zhang et al., 2018) and forms a covalently closed cyclic structure (Li et al., 2018a). CircRNAs have been found to participate in the progression of multiple pathophysiological processes, including cancers (Kristensen et al., 2018; Patop and Kadener, 2018; Di Agostino et al., 2020). They play critical roles in many biological processes of tumorigenesis, such as cell viability (Chen et al., 2017), invasion (Song et al., 2019), migration (Ren et al., 2019), and angiogenesis (Jia et al., 2016). Recently, the role of circRNAs in gliomagenesis progression has also been studied. Yang et al. (2018) found that circRNA FBXW7 suppresses glioma tumorigenesis, Xiong et al. (2019) revealed that circRNA circMAN2B2 mediates glioma progression by regulating S100A8 expression through sponging of miR-1205. Barbagallo et al. (2018) demonstrated that circSMARCA5 affects the migration of glioblastoma multiforme cells by facilitating SRSF1/SRSF3/PTB axis activity. These studies suggest an essential role of circRNA in glioma progression.

CircRNA circTLK1, derived from TLK1 messenger RNA (mRNA), was first identified in a renal cell carcinoma study as an oncogene. CircTLK1 upregulates CBX4 expression to promote renal cell carcinoma development by acting as a molecular sponge for miR-136-5p (Li et al., 2020). Subsequently, Wu F. et al. (2019) revealed the promotive effects of circTLK1 in ischemic stroke. The function of circTLK1 in myocardial ischemia/reperfusion injury was demonstrated by Song Y.F. et al. (2020). However, whether circTLK1 participates in glioma progression remains unclear.

Here, we aimed to uncover the role of circTLK1 in the initiation and progression of glioma. We found that circTLK1 expression is upregulated in glioma tissues and cell lines. Furthermore, downregulation of circTLK1 inhibited glioma cellular progress and suppressed cell growth *in vivo*. Investigation of the mechanisms upstream and downstream of circTLK1 revealed that circTLK1 mediated by PBX2 aggravates glioma progression by activating JAK/STAT signaling via the miR-452-5p/SSR1 axis.

## Materials and Methods

### Clinical Samples

Thirty pairs of glioma tissues and normal marched tissues were harvested from patients who were diagnosed with glioma in Huai’an Hospital Affiliated to Xuzhou Medical University, Second People’s Hospital of Huai’an City from January 2017 to June 2018 and were stored at −80°C. These patients did not receive radiotherapy or chemotherapy. Informed consent was obtained from all patients. Sample status was confirmed by two pathologists. This study was approved by the Ethics Committee of the Huai’an Hospital Affiliated to Xuzhou Medical University, Second People’s Hospital of Huai’an City (No. HEYLL 201928).

### Cell Culture and Treatment

Glioma cell lines (T98G, LN229, CRT, U251, M059J, and M059K), normal human astrocytes (NHAs), and 293T cells were purchased from the Cell Bank of Shanghai Institutes of Biological Sciences, Chinese Academy of Sciences (Shanghai, China). Cells were maintained in Dulbecco’s modified Eagle’s medium (DMEM) (Invitrogen, Carlsbad, CA, United States) with 10% FBS (Gibco, Waltham, MA, United States) in a 37°C, 5% CO_2_ environment. All plasmids, Sh-NC, Sh-circTLK1#1, Sh-circTLK1#2, OE-NC, and OE-circTLK1#1, were purchased from RiboBio (Guangzhou, China). All transfections were performed using Lipofectamine 2000 (Invitrogen, Carlsbad, CA, United States) following the manufacturer’s instructions.

### Animal Experiment

Eight-week-old nude mice were separated into three groups (*n* = 6), subcutaneously inoculated with U251 cells (10^6^ per mouse), which were preinfected with Sh-NC, Sh-circTLK1#1, and Sh-circTLK1#2. After 21 days, tumor samples from mice were harvested. Tumor volumes or end weights were recorded. The animal experiments were approved by the Ethics Committee of the Huai’an Hospital Affiliated to Xuzhou Medical University, Second People’s Hospital of Huai’an City.

### Quantitative Real-Time Polymerase Chain Reaction

All RNAs from cells or human tissues were isolated using TRIzol reagent (Invitrogen, Carlsbad, CA, United States). The PARIS^TM^ Kit (Invitrogen, Carlsbad, CA, United States) was use to perform nuclear and cytoplasmic RNA fractionation. The Reverse Transcription Kit (Invitrogen, Carlsbad, CA, United States) was used to reverse transcribe circRNA, mRNA, and RNA into complementary DNA (cDNA). An internal reference was applied using glyceraldehyde-3-phosphate dehydrogenase (GAPDH). Quantitative real-time PCR (qRT-PCR) assays were conducted using the SYBR-Green Real-Time Kit (Takara, Tokyo, Japan) on a Bio-Rad CFX96 system. The fold expression changes were analyzed using the 2^–ΔΔCt^ method. All primers used in the current study were as follows: circTLK1, F, 5′-ACAGTTTTGGAAGCTTGGGATCT-3′ and R, 5′-TGCTCCCACTTGCAACTCCA3′; miR-452-5p, F, 5′-TCGGCAATCATGATGGGCTCCTC-3′ and R, 5′-CTCA ACTGGTGTCGTGGAGTC-3′; SSR1, F, 5′-AAGAACTACAAA ACCGCCCC-3′ and R, 5′-ATCCCAGGCTGAGACCCAT-3′; PBX2, F, 5′-CCCATGTCATGAACCTGCTG-3′ and R, 5′-GC GCTGAACTTTCGATGGAT-3′; GAPDH, F, 5′-AAGGT CGGAGTCAACGGATTT-3′ and R, 5′-ACCAGAGTTAAAAG CAGCCCTG-3′.

### Fluorescence *in situ* Hybridization Assay

The Cy3-labeled circTLK1 probe was synthesized and commercially obtained from RiboBio (Guangzhou, China) to localize circTLK1 expression in NHAs. A fluorescence *in situ* hybridization kit (Geneseed, Guangzhou, China) was used to perform fluorescence *in situ* hybridization (FISH) assay following the manufacturer’s instructions. The cells were imaged using a fluorescence microscope (Leica, Wetzlar, Germany).

### Western Blot

All proteins were isolated using radioimmunoprecipitation assay lysis buffer (Beyotime, Shanghai, China). Bicinchoninic acid (BCA) (Beyotime, Shanghai, China) was used to quantify the proteins. The proteins were separated using 10% sodium dodecyl sulfate–polyacrylamide gel electrophoresis (SDS-PAGE) and transferred onto a polyvinylidene difluoride membrane (EMD Millipore, Billerica, MA, United States). The membranes were blocked with 5% skim milk at 37°C for 90 min. Next, membranes were incubated at 4°C overnight with primary antibodies as follows: JAK1 (CST; 1:1,000; 29261S), p-JAK1 (CST; 1:1,000; 74129S), STAT1 (CST; 1:1,000; 14994S), p-STAT1 (CST; 1:1,000; 9167S), STAT3 (CST; 1:1,000; 8768S), p-STAT3 (CST; 1:2,000; 9145S), SSR1 (Sigma; 0.4 μg/ml; HPA017062), and GAPDH (CST; 1:1,000; 5174S). Subsequently, the membranes were washed with Tris-buffered saline with Tween 20 (TBST) and incubated with secondary antibodies for 2 h. Protein bands were visualized using ECL (Beyotime, Shanghai, China).

### Hematoxylin and Eosin Staining

Mouse tumor tissues were immersed in 10% neutral formalin for 1 day under sterile conditions. Next, the tumor samples were dehydrated, embedded in paraffin, and cut into 4-μm-thick sections. H&E staining was performed via a standard procedure.

### CCK-8 Assay

Cell proliferation assays were conducted using a cell counting kit 8 (CCK-8) solution (Dojindo, Kumamoto, Japan). Cells were cultured in a 96-well plate and incubated with the solution for 3 h. Absorbance at 450 nm was measured using a Thermomax microplate reader (Molecular Devices, CA, United States). All assays were performed at least three times.

### Transwell Assay

Cell migration was measured using a Transwell chamber (Corning Inc., Corning, NY, United States) with an 8.0-μm pore size polycarbonate membrane. Cells (10^5^ per chamber) were cultured in up-chambers (matrix for invasion assay) for 24 h, after which cells were removed from the chamber. The cells on the reverse side of the chamber were fixed using 4% paraformaldehyde and then stained with crystal violet. The migrated cells were visualized using an IX71 inverted microscope (Olympus, Tokyo, Japan).

### Scratch Wound Healing Assay

U251 and CRT cells, upon indicated transfections, were seeded in a six-well plate for culturing at 37°C in a 5% CO_2_ environment. The fine end of a 10-μl pipette tip was used to create a scratch wound. The cells were imaged using phase-contrast microscopy at the indicated times.

### Biotinylated RNA Pull-Down

Biotinylated circTLK1 probes, miR-452-5p probes, and normal control probes were obtained from RiboBio (Guangzhou, China). Coimmunoprecipitation buffer (Beyotime) was used to lyse the cells. Probe-coated beads were constructed using the coincubating biotinylated probe and C-1 magnetic beads (Life Technologies, CA, United States) for 120 min. The probes were then incubated with cell lysates overnight. TRIzol reagent was used for RNA isolation, and qRT-PCR assays were performed to analyze the RNA complexes.

### AGO2-RNA Binding Protein Immunoprecipitation

RIP assays were performed using a Magna RIP RNA-binding protein immunoprecipitation kit (Millipore, Germany) with anti-AGO2 and anti-IgG following the manufacturer’s instructions. The complexes were evaluated using qRT-PCR assays.

### Chromatin Immunoprecipitation

First, glycine was used to terminate the crosslinking reaction in cells with 1% formaldehyde. Subsequently, the bound RNA-protein was subjected to sonication for fragment production. Next, the antibodies were added, and the proteinated A-Sepharose beads were applied to the immunoprecipitated fractions. The results were analyzed using qRT-PCR analysis.

### Luciferase Reporter Assays

The wild-type (WT) or mutant-type (Mut) sequences of circTLK1, circTLK1 promoter, or SSR1 were subjected to PmirGLO dual-luciferase vectors and then transfected into U251 and 293T cells with NC mimics miR-452-5p mimics or PcDNA 3.1/PcDNA 3.1-PBX2. After 24 h, the luciferase activities in U251 or 293T cells were detected using a dual-luciferase reporter assay system (Promega, WI, United States). Firefly luciferase activity was normalized to Renilla luciferase activity.

### Statistical Analysis

Statistical analyses were performed using the SPSS 20 software (SPSS, Chicago, IL, United States). Results are shown as mean ± SD. Results in two different groups were calculated using unpaired Student’s *t-*test, and results among three or more groups were analyzed using one-way ANOVA. All assays were conducted at least thrice. Spearman analysis was used to evaluate the association of expression in the samples. Statistical significance was set at *p* < 0.05.

## Results

### Measurement of circTLK1 in Gliomas

**FIGURE 1:**
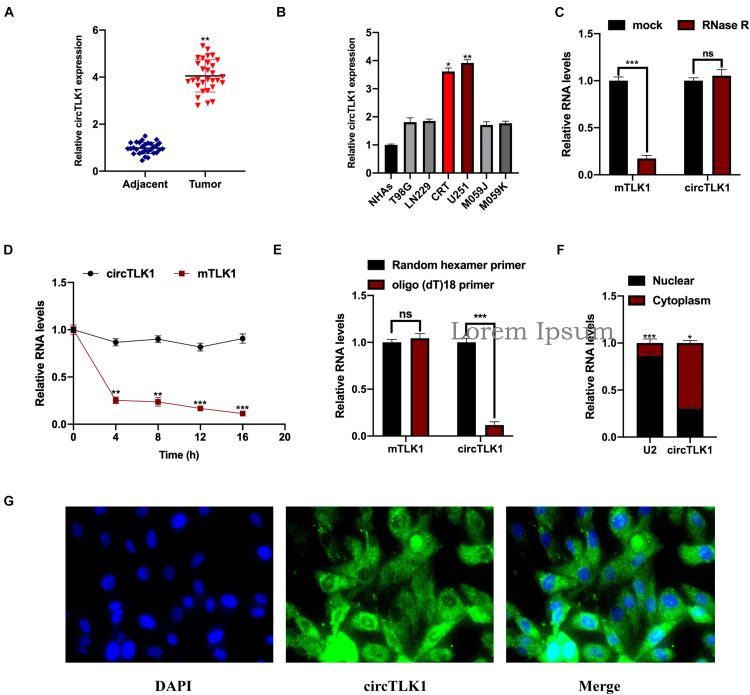
Measurement of circTLK1 in gliomas. **(A)** Determination of the circTLK1 expression in 30 pairs of glioma tumor tissues and matched normal tissues using qRT-PCR. **(B)** qRT-PCR was used to measure circTLK1 expression in glioma cells T98G, LN229, CRT, U251, M059J, M059K, and normal human astrocytes NHAs. **(C)** Relative RNA levels were analyzed using RT-qPCR; the mock group was used for internal normalization. **(D)** U251 cells were treated with the transcription inhibitor actinomycin D to evaluate mTLK1 and circTLK1 stability. **(E)** Relative RNA levels analyzed using RT-qPCR in U251 cells; random hexamer primers were used for internal normalization. **(F)** The level of circTLK1 in the nuclear and cytoplasm of NHAs was measured using a nuclear-cytoplasmic fractionation assay. **(G)** FISH analysis was conducted to detect the distribution of circTLK1 in NHAs. *p < 0.05, **p < 0.01, ***p < 0.001.

First, to uncover the role of circTLK1 in glioma, we assessed circTLK1 expression levels in 30 pairs of human glioma tissues. It was found that circTLK1 expression in glioma tumors was markedly higher than in normal matched tissues (Figure 1A). As shown in Figure 1B, circTLK1 expressed in T98G, LN229, CRT, U251, M059J, and M059K cells was higher than that in NHAs, and circTLK1 was abundantly expressed in U251 and CRT cells. Furthermore, we assessed the circRNA characteristics of circTLK1. The linear TLK1 (mTLK1) expression was significantly downregulated in U251 and CRT cells treated with RNase R; however, the expression of circTLK1 remained stable (Figure 1C). Subsequently, circTLK1 was found to be more stable than mTLK1 in U251 cells treated with the transcription inhibitor actinomycin D (Figure 1D). Random hexamer and oligo (dT)18 primers were used to amplify the TLK1 RNAs. As shown in Figure 1E, TLK1 mRNA expression was significantly upregulated but not that of the circular RNA. Moreover, nuclear-cytoplasmic fractionation assays (Figure 1F) and FISH analysis (Figure 1G) revealed that circTLK1 was primarily distributed in the cytoplasm. These findings suggests that circTLK1 may be involved in glioma development.

### Downregulated circTLK1 Inhibits Glioma Cell Proliferation, Migration, and Invasion

**FIGURE 2:**
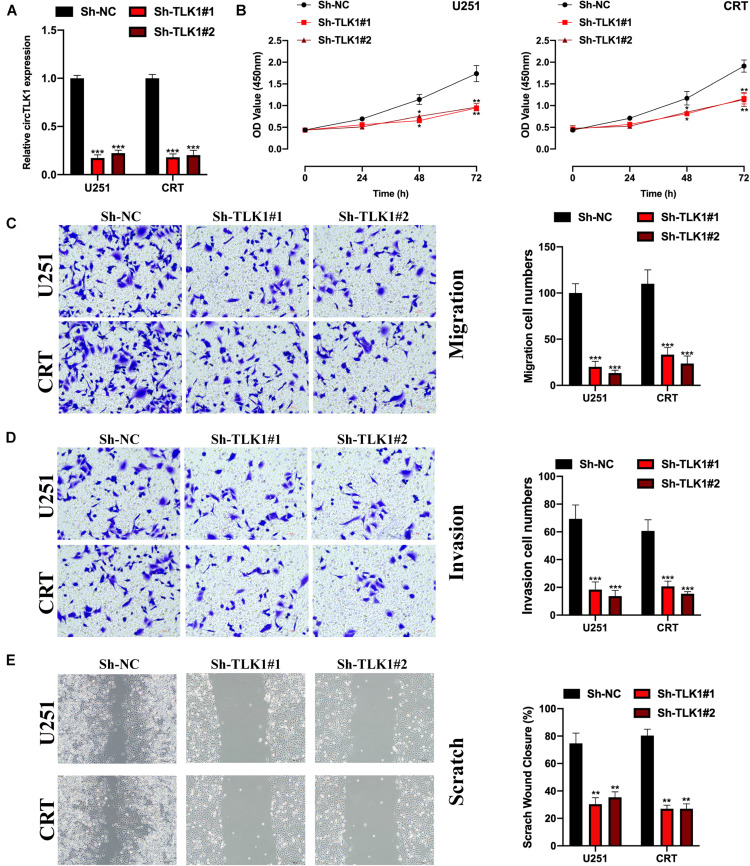
Downregulated circTLK1 inhibits glioma cell proliferation, migration, and invasion. **(A)** Transfection efficiencies of Sh-NC, Sh-circTLK1#1, Sh-circTLK1#2 in U251, and CRT detected using qRT-PCR. **(B)** CCK-8 assay assessing the cell proliferation abilities. **(C)** Transwell migration assay was performed to measure the cell migration levels. **(D)** Transwell invasion experiment was applied to detect cell invasion abilities. **(E)** Scratch test was applied to evaluate the migration ability of transfected cells. Comparative statistics are shown here. **p < 0.01, ***p < 0.001.

Here, we aimed to uncover the biological function of circTLK1 in glioma progression. Sh-NC, Sh-circTLK1#1, and Sh-circTLK1#2 were constructed and transfected into U251 and CRT cells. The relative expression of circTLK1 in U251 and CRT cells was measured (Figure 2A), and it was found that downregulated circTLK1 significantly inhibited cell proliferation (Figure 2B). Furthermore, our results showed that downregulated circTLK1 suppressed glioma cell migration and invasion (Figures 2C–E). Taken together, the results show that circTLK1 was involved in glioma progression by mediating increased cell viability, migration, and invasion of glioma cells *in vitro*.

### Downregulated circTLK1 Inhibits Glioma Cell Growth *in vivo*

**FIGURE 3:**
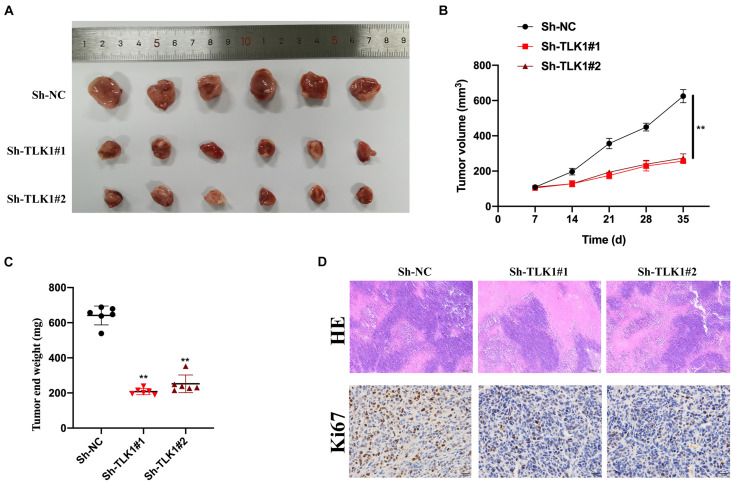
Downregulated circTLK1 inhibits glioma cell growth *in vivo*. Mice were separated into three groups (*n* = 6) and then subcutaneously injected with U251 (10^6^ per mouse) cells preinfected with Sh-NC, Sh-circTLK1#1, and Sh-circTLK1#2. **(A)** Representative image of subcutaneously incubated tumors (*n* = 6). **(B)** Tumor volumes were recorded every 3 days, and statistical results are presented. **(C)** Tumor end weights were recorded, and comparative statistics are shown. **(D)** Tumor histology was assessed using H&E staining (scale bar, 200 μm), and the proliferation marker Ki67 was detected using IHC staining (scale bar, 50 μm). **p < 0.01.

Next, we evaluated the function of circTLK1 in a xenograft nude mouse model. U251 cells (10^6^ per mouse), which were preinfected with Sh-NC, Sh-circTLK1#1, and Sh-circTLK1#2, were injected into nude mice. As shown in Figure 3A, representative images of subcutaneous tumors indicated that knockdown of circTLK1 suppressed tumor growth *in vivo*. Tumor weights and volumes were recorded, and comparative statistics were analyzed (Figures 3B,C). HE staining assays were performed, and the expression of proliferation biomarker Ki67 was assessed in mouse tumor tissues by immunohistochemistry (IHC) staining. The level of Ki67 was obviously decreased in circTLK1 knockdown tumors (Figure 3D). These results indicated that downregulation of circTLK1 suppressed glioma cell growth *in vivo*.

### circTLK1 Modulates Gliomagenesis via Activating JAK/STAT Signaling

**FIGURE 4:**
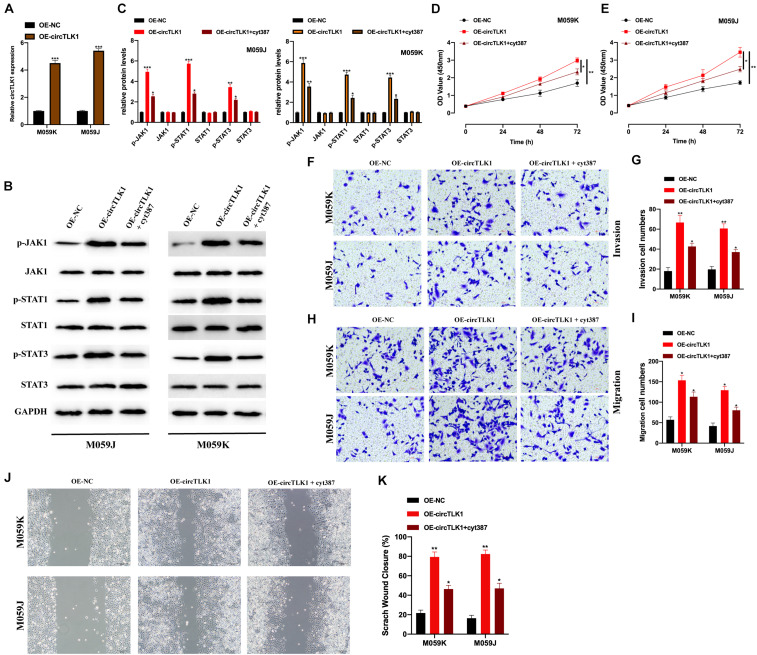
CircTLK1 modulates gliomagenesis by activating JAK/STAT signaling. M059K and M059J cells were transfected with OE-NC or OE-circTLK1. **(A)** M059K and M059J cells were subjected to qRT-PCR to detect transfection efficiencies. **(B,C)** The expression of JAK/STAT pathway proteins in M059K and M059J cells, which were pretransfected with OE-NC, OE-circTLK1, and JAK/STAT inhibitor cyt387 as indicated, measured using Western blot, and comparative statistics are presented. **(D,E)** CCK-8 assays were conducted to measure the proliferation levels. **(F,G)** Treated M059K and M059J cells were subjected to a Transwell migration experiment to measure the cell migration level. **(H,I)** Transwell invasion assays were applied to detect cell invasion levels of treated M059K and M059J cells. **(J,K)** Scratch test was applied to evaluate the migration ability of transfected cells. *p < 0.05, **p < 0.01, ***p < 0.001.

Previous studies have suggested that JAK/STAT signaling plays a role in glioma progression (Tu et al., 2011; Zhang et al., 2019; Swiatek-Machado and Kaminska, 2020). To explore whether circTLK1 regulates glioma progression through JAK/STAT signaling, we constructed OE-NC and OE-circTLK1 and transfected them into M059K and M059J cells. CircTLK1 expression was measured in the transfected cells (Figure 4A). Western blot analysis revealed that p-JAK1, p-STAT1, and p-STAT3 expression were significantly increased upon circTLK1 overexpression, while JAK1, STAT1, and STAT3 expression remained unchanged (Figures 4B,C). Moreover, glioma cell models were generated by transfecting OE-NC, OE-circTLK1, and JAK/STAT inhibitor cyt387 as indicated. Overexpression of circTLK1 in M059K and M059J cells promoted cell proliferation (Figures 4D,E), migration (Figures 4F,G,J,K), and invasion (Figures 4H,I), but these phenomena were rescued by treatment with the JAK/STAT inhibitor cyt387. The above results indicate that circTLK1 promotes gliomagenesis by regulating JAK/STAT signaling.

### circTLK1 Regulates SSR1 Expression Through Sponging miR-452-5p

**FIGURE 5:**
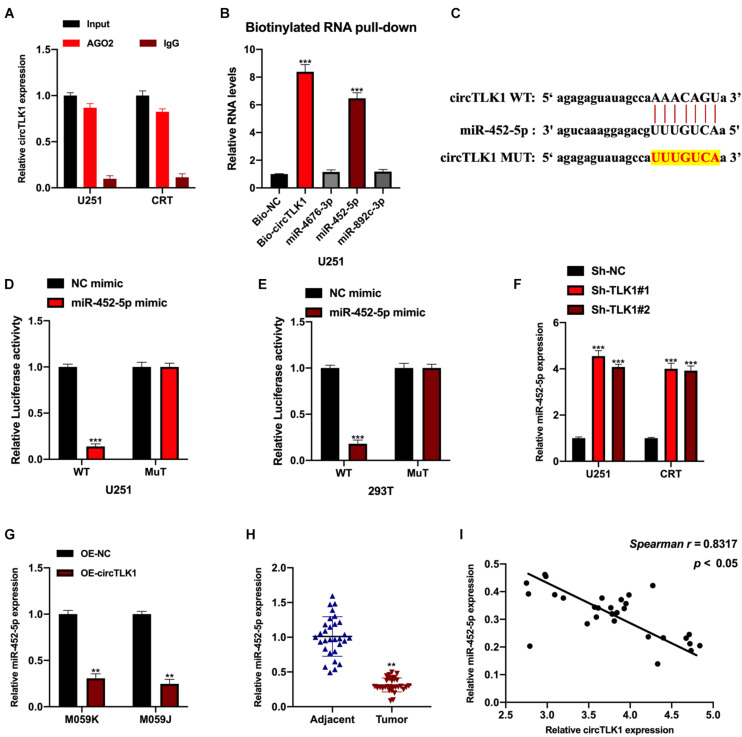
CircTLK1 sponges to miR-452-5p. ENCORI database (http://starbase.sysu.edu.cn/index.php) with CLIP data, strict stringency (≥ 5), and degradome data, medium stringency (≥2) **(A)** mRNA binding ability of circTLK1 was detected using AGO2-RIP assays in U251 cells. **(B)** qRT-PCR was performed to assess relative RNA expressions in U251 cells. **(C)** The binding sites between miR-452-5p and circTLK1 (WT or MUT). **(D,E)** Relative luciferase activities in NC mimic, miR-452-5p mimic, and reporter vectors containing circTLK1 WT and Mut sequences transfected into 293T and U251 cells were measured. **(F)** Relative miR-452-5p expression in U251 and CRT cells pretreated with Sh-NC, Sh-circTLK1#1, and Sh-circTLK1#2 was measured using qRT-PCR. **(G)** miR-452-5p expression in U251 and CRT cells pretreated with OE-NC and OE-circTLK1 was evaluated using qRT-PCR. **(H)** The miR-452-5p expression level in glioma tissues was detected using qRT-PCR. **(I)** The circTLK1 and miR-452-5p expression association was calculated using Spearman analysis. **p < 0.01, ***p < 0.001.

First, we assessed the mRNA binding ability of circTLK1 in U251 cells. AGO2-RNA binding protein immunoprecipitation (AGO2-RIP) assays were performed, and it was found that circTLK1 was abundantly enriched in AGO2 antibody complexes in comparison with anti-IgG (Figure 5A), which showed that circTLK1 acts as a competitive endogenous RNA (ceRNA) in gliomagenesis. Then, to understand the mechanisms underlying circTLK1 in gliomagenesis, downstream factors were investigated via bioinformatics analysis. Three putative mRNAs were selected, and biotinylated RNA pull-down assays were performed. As shown in Figure 5B, in comparison with bio-NC, miR-452-5p expression was found to be significantly higher than that of other putative mRNAs, suggesting that miR-452-5p might bind to circTLK1. The predicted binding sites between miR-452-5p and circTLK1 (WT or MUT) were synthesized (Figure 5C). The correlation between miR-452-5p and circTLK1 was assessed using a luciferase reporter gene assay. Luciferase activities in 293T and U251 cells preinfected with miR-452-5p mimic and vectors harboring circTLK1 WT sequences were evidently decreased (Figures 5D,E). Subsequently, miR-452-5p expression levels in U251 and CRT cells, infected with Sh-NC, Sh-circTLK1#1, and Sh-circTLK1#2, were detected. Knockdown of circTLK1 upregulated miR-452-5p expression (Figure 5F). Moreover, miR-452-5p expression was inhibited by circTLK1 (Figure 5G) and downregulated in glioma tumor tissues (Figure 5H). The miR-452-5p expression in glioma tumor tissues was significantly correlated with circTLK1 (Figure 5I).

**FIGURE 6:**
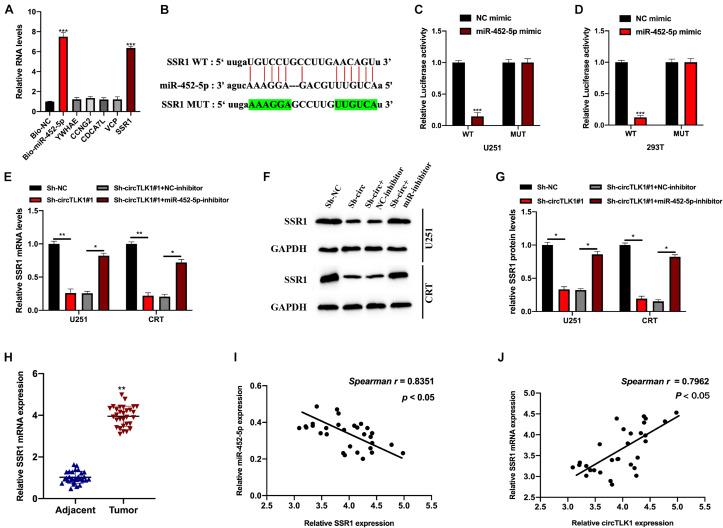
CircTLK1 regulates SSR1 expression through sponging miR-452-5p. PITA database (http://genie.weizmann.ac.il/pubs/mir07/mir07_dyn_data.html) and DIANA-microT (http://diana.imis.athena-innovation.gr/DianaTools/index.php?r=microT_CDS/index) with CLIP Data, strict stringency (≥5), and degradome data, high stringency (≥3), was used. **(A)** qRT-PCR was used to assess potential binding mRNAs expression and bio-miR-452-5p expression in U251 cells. **(B)** The WT and MUT binding sites between SSR1 and miR-452-5p. **(C,D)** Relative luciferase activities in NC mimic, miR-452-5p mimic, and reporter vectors containing SSR1 WT and Mut sequences transfected into 293T and U251 cells were measured. **(E–G)** The protein and mRNA expressions of SSR1 in U251 and CRT pretreated with Sh-NC, Sh-TLK1#1, Sh-TLK1#1 + NC-inhibitor, and Sh-TLK1#1 + miR-452-5p-inhibitor were measured using qRT-PCR and Western blot. All experiments were performed in triplicates. **(H)** SSR1 expression in glioma tissues was assessed using qRT-PCR. **(I,J)** Spearman analysis was used to calculate the association between SSR1 and circTLK1 or miR-452-5p. *p < 0.05, **p < 0.01, ***p < 0.001.

Next, we explored the target mRNA of miR-452-5p using the PITA and DIANA-microT databases. The expression of potential mRNA targets in biotinylated probes transfected into U251 cells was assessed; the SSR1 expression level was significantly higher in these cells than in the other groups (Figure 6A). The binding sites between SSR1 (WT or MUT) and miR-452-5p are presented in Figure 6B. Subsequently, we found that luciferase activity in 293T and U251 cells preinfected with miR-452-5p mimic and a vector containing SSR1 WT sequences were markedly decreased (Figures 6C,D). The SSR1 expression level was significantly inhibited by circTLK1 knockdown but reversed by miR-452-5p inhibition (Figures 6E–G). This indicated that SSR1 was a downstream target of miR-452-5p and positively mediated by circTLK1. Furthermore, we found that SSR1 expression in glioma tumor tissues was downregulated and correlated with miR-452-5p or circTLK1 expression (Figures 6H–J).

### circTLK1 Promotes Gliomagenesis via miR-452-5p/SSR1/JAK/STAT Pathway

**FIGURE 7:**
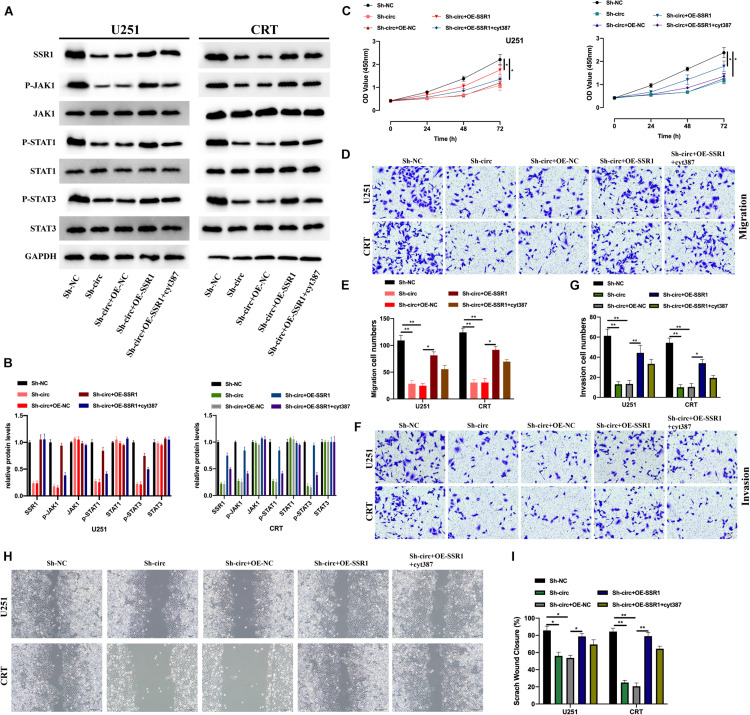
CircTLK1 promotes gliomagenesis via the miR-452-5p/SSR1/JAK/STAT pathway. **(A,B)** The expressions of SSR1 and JAK/STAT pathway proteins in Sh-NC, Sh-circTLK1#1, Sh-circTLK1#1 + OE-NC, Sh-circTLK1#1 + OE-SSR1, and Sh-circTLK1#1 + OE-SSR1 + cyt387 transfected into U251 and CRT cells were detected using Western blot, and comparative statistics were analyzed. **(C)** CCK-8 was used to measure the proliferation levels. **(D,E)** Transwell migration assay was applied to measure migration abilities of treated U251 and CRT cells. **(F,G)** Transwell invasion assay was applied to assess cell invasion abilities of treated U251 and CRT cells, and comparative statistics are presented. **(H,I)** The migration level of transfected cells was detected using the scratch test, and results were analyzed as indicated. *p < 0.05 and **p < 0.01.

We further explored whether circTLK1 mediates JAK/STAT signaling via the miR-452-5p/SSR1 axis. For this purpose, we constructed glioma knockdown cell models and evaluated the expression of SSR1, and JAK/STAT pathway proteins in these cell models were measured (Figures 7A,B). Subsequently, the biological functions of each cell model were assessed. It was found that overexpression of SSR1 inhibited the suppressive effects of Sh-circTLK1 on cell proliferation (Figure 7C), migration (Figures 7D,E,H,I), and invasion (Figures 7F,G), but these phenomena were rescued by treatment with the JAK/STAT inhibitor cyt387. This suggested that circTLK1/miR-452-5p/SSR1 participates in glioma progression by mediating JAK/STAT signaling.

### circTLK1 Expression Is Mediated by PBX2

**FIGURE 8:**
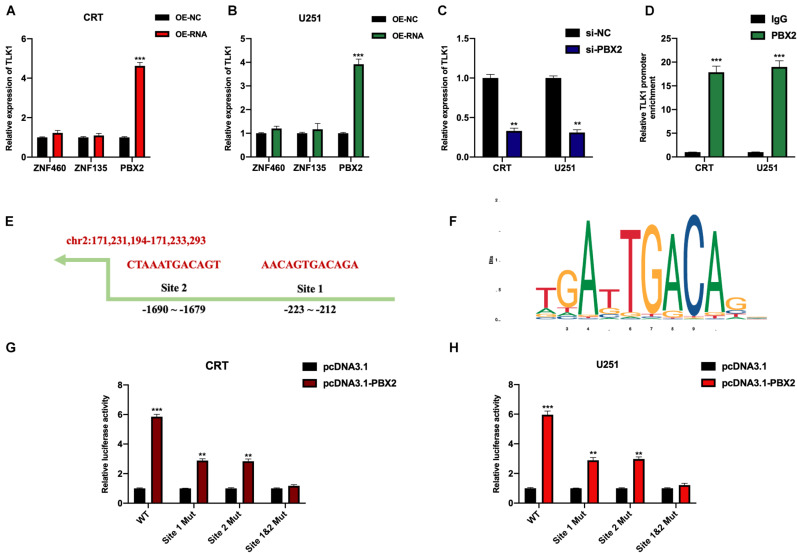
CircTLK1 expression is mediated by PBX2. CRT and U251 cells were stably infected with OE-NC, OE-RNAs, si-NC, and si-PBX2, as indicated. **(A,B)** Relative expression of circTLK1 in **(A)** CRT and **(B)** U251 cells pretreated with OE-NC and OE-RNAs was measured using qRT-PCR. **(C)** Relative expression of circTLK1 in CRT and U251 cells pretransfected with si-NC and si-PBX2 was detected using qRT-PCR. **(D)** ChIP assay was performed using anti-IgG and anti-PBX2; results were detected using the qRT-PCR assay. **(E)** The predicted binding sites of circTLK1 promoter were obtained from the JASPAR dataset. **(F)** The predicted binding sequence of PBX2 was collected from the JASPAR dataset. **(G,H)** The impact of PBX2 on circTLK1 transcription in **(G)** CRT and **(H)** U251 cells was assessed using luciferase reporter assay. **p < 0.01, ***p < 0.001.

CircRNA expression could be regulated by transcriptional factors (Wang et al., 2018; Wu L. et al., 2019; Lv et al., 2020), which is an important mechanism in circRNA functional patterns. Here, we further investigated the mechanisms upstream of circTLK1 in gliomagenesis. Using the JASPAR^1^ dataset, we identified three transcriptional regulators (ZNF460. ZNF135, and PBX2) that may mediate circTLK1 expression. Subsequently, we constructed dysregulation cell models of each regulator and measured the expression levels of circTLK1 in these cells. As shown in Figures 8A–C, circTLK1 expression was upregulated in pre-B-cell leukemia transcription factor 2 (PBX2)-overexpressing cells and downregulated in PBX2 knockdown cells. Furthermore, chromatin immunoprecipitation (ChIP) assay results showed that TLK1 was markedly enriched after treatment with anti-PBX2 antibody as to isotype control (Figure 8D). Our results suggest that PBX2 might be at functional upstream regulator of circTLK1 in glioma cells. The predicted binding sequences between the TLK1 promoter and PBX2 were obtained from the JASPAR dataset (Figures 8E,F). The interaction between the TLK1 promoter and PBX2 was assessed using a luciferase reporter assay. As shown in Figures 8G,H, PBX2 overexpression promoted the activity of the wild type (WT) of the TLK promoter, while this phenomenon was attenuated in each mutant type (Mut) of the TLK promoter. Furthermore, the promotive effect of PBX2 overexpression on the TLK1 promoter was completely abrogated when the site 1 and 2 binding sequences were mutated. Our results indicated that circTLK1 expression in glioma cells could be mediated by the transcriptional regulator PBX2.

## Discussion

The findings of this study revealed that circTLK1 mediated by PBX2 regulated JAK/STAT signaling to promote glioma development by facilitating miR-452-5p/SSR1. First, it was found that that circTLK1 was expressed at higher levels in glioma tissues than in matched normal tissues. The circular RNA characteristic of circTLK1 was assessed. CircTLK1 is abundantly expressed in glioma cells. These results suggest that circTLK1 may be involved in glioma development.

Subsequently, the biological functions of circTLK1 in glioma cell lines were assessed. It was found that circTLK1 knockdown suppressed glioma cell progression by performing CCK-8, Transwell, and scratch assays. Then, animal models were constructed, and our results suggested that downregulated circTLK1 suppressed tumor cell growth *in vivo*. We noticed that JAK/STAT signaling plays a crucial role in glioma progression, and we presumed that circTLK1 plays a role in mediating JAK/STAT signaling in glioma progression. The expressions of p-JAK1, p-STAT1, and p-STAT3 in circTLK1-overexpressing cells were upregulated, suggesting that JAK/STAT signaling was activated by circTLK1. Moreover, the promotive effects of circTLK1 on glioma cellular progress were rescued by treatment with the JAK/STAT signaling inhibitor cyt387. Our results imply that circTLK1 participates in glioma progression by activating JAK/STAT signaling.

Previous studies have demonstrated that miR-452-5p participates in multiple biological processes, such as colorectal cancer (Yan et al., 2020b), gastric cancer (Zhu et al., 2020), hepatocellular cancer (Yang et al., 2020), prostate cancer (Song X. et al., 2020), and renal cancer (Zhai et al., 2018). However, the role of miR-452-5p in gliomas has not been previously reported. Our results revealed that circTLK1 sponges miR-452-5p and negatively mediates miR-452-5p expression. Signal sequence receptor subunit 1 (SSR1) was found to be a downstream target of miR-452-5p in glioma cells. SSR1 was found to be associated with hypopharyngeal squamous cell carcinoma (Yan et al., 2020a) and breast cancer (Funakoshi et al., 2019). However, whether SSR1 participates in glioma progression remains unclear. Our results revealed that miR-452-5p directly targeted SSR1 and suppressed its expression in glioma cells. Furthermore, JAK/STAT signaling was found to be mediated by the circTLK1/miR-452-5p/SSR1 axis.

The expression of circRNAs can be regulated by transcription factors. Lv et al. (2020) demonstrated that circ-MMP2 expression in lung adenocarcinoma cells is induced by FOXM1 and that the transcription factor c-FOS could bind to the promoter region of circPVT1 and promote circPVT1 expression in non-small cell lung cancer cells (Li et al., 2018b). Wang et al. (2018) demonstrated that circ-4099 expression in intervertebral disk degradation is regulated by TNF-α-induced GRP78. Transcription factor-induced expression is an important pattern of circRNAs in various biological processes. In this study, we have partially revealed the downstream mechanisms affected by circTLK1 in gliomagenesis, and we further investigated the upstream regulator of circTLK1. By utilizing the JASPAR dataset, ChIP, and luciferase reporter assays, we identified that PBX2 could bind to the circTLK1 promoter and mediate circTLK1 expression in glioma cells.

Although we partially demonstrated the existence of the novel PBX2/circTLK1/miR-452-5p/SSR1 axis in glioma progression, this will require extensive investigation. Our clinical results require additional samples for further confirmation. Moreover, the relationship between SSR1 and JAK/STAT signaling needs to be verified.

In conclusion, we partially elucidated the role of circTLK1 in glioma progression. CircTLK1 mediated by PBX2 regulates JAK/STAT signaling to promote glioma development via the miR-452-5p/SSR1 axis. Our results provide novel diagnostic and therapeutic targets for treating glioma.

## Data Availability Statement

The original contributions presented in the study are included in the article/supplementary material, further inquiries can be directed to the corresponding author/s.

## Ethics Statement

The studies involving human participants were reviewed and approved by the Ethics Committee of the Huai’an Hospital Affiliated to Xuzhou Medical University. The patients/participants provided their written informed consent to participate in this study. The animal study was reviewed and approved by the Ethics Committee of the Huai’an Hospital Affiliated to Xuzhou Medical University. Written informed consent was obtained from the individual(s) for the publication of any potentially identifiable images or data included in this article.

## Author Contributions

JL, YW, and RY: conception, design, supervision, resources, manuscript writing, and revising. ZZ and XW: experiments conduction and data analysis. QM and HJ: data visualization and statistical analysis. All authors contributed to the article and approved the submitted version.

## Conflict of Interest

The authors declare that the research was conducted in the absence of any commercial or financial relationships that could be construed as a potential conflict of interest.

## Publisher’s Note

All claims expressed in this article are solely those of the authors and do not necessarily represent those of their affiliated organizations, or those of the publisher, the editors and the reviewers. Any product that may be evaluated in this article, or claim that may be made by its manufacturer, is not guaranteed or endorsed by the publisher.

## Footnotes

1
http://jaspar.genereg.net/

## References

[B1] Barbagallo D. Caponnetto A. Cirnigliaro M. Brex D. Barbagallo C. D’Angeli F. (2018). CircSMARCA5 inhibits migration of glioblastoma multiforme cells by regulating a molecular axis involving splicing factors SRSF1/SRSF3/PTB. *Int. J. Mol. Sci.* 19:480. 10.3390/ijms19020480 29415469 PMC5855702

[B2] Chen G. Shi Y. Zhang Y. Sun J. (2017). CircRNA_100782 regulates pancreatic carcinoma proliferation through the IL6-STAT3 pathway. *Onco Targets Ther.* 10 5783–5794. 10.2147/ott.s150678 29255366 PMC5722018

[B3] Chen Y. Xu R. (2016). Drug repurposing for glioblastoma based on molecular subtypes. *J. Biomed. Inform.* 64 131–138. 10.1016/j.jbi.2016.09.019 27697594 PMC6146394

[B4] Di Agostino S. Riccioli A. De Cesaris P. Fontemaggi G. Blandino G. Filippini A. (2020). Circular RNAs in embryogenesis and cell differentiation with a focus on cancer development. *Front. Cell Dev. Biol.* 8:389. 10.3389/fcell.2020.00389 32528957 PMC7266935

[B5] Funakoshi Y. Wang Y. Semba T. Masuda H. Hout D. Ueno N. T. (2019). Comparison of molecular profile in triple-negative inflammatory and non-inflammatory breast cancer not of mesenchymal stem-like subtype. *PLoS One* 14:e0222336. 10.1371/journal.pone.0222336 31532791 PMC6750603

[B6] Jia P. Cai H. Liu X. Chen J. Ma J. Wang P. (2016). Long non-coding RNA H19 regulates glioma angiogenesis and the biological behavior of glioma-associated endothelial cells by inhibiting microRNA-29a. *Cancer Lett.* 381 359–369. 10.1016/j.canlet.2016.08.009 27543358

[B7] Khasraw M. Ameratunga M. S. Grant R. Wheeler H. Pavlakis N. (2014). Antiangiogenic therapy for high-grade glioma. *Cochrane Database Syst. Rev.* 9 CD008218. 10.1002/14651858.CD008218.pub3 25242542

[B8] Kristensen L. S. Hansen T. B. Veno M. T. Kjems J. (2018). Circular RNAs in cancer: opportunities and challenges in the field. *Oncogene* 37 555–565. 10.1038/onc.2017.361 28991235 PMC5799710

[B9] Li J. Huang C. Zou Y. Ye J. Yu J. Gui Y. (2020). CircTLK1 promotes the proliferation and metastasis of renal cell carcinoma by sponging miR-136-5p. *Mol. Cancer* 19:103. 32503552 10.1186/s12943-020-01225-2 PMC7275467

[B10] Li X. Yang L. Chen L. L. (2018a). The biogenesis, functions, and challenges of circular RNAs. *Mol. Cell* 71 428–442. 10.1016/j.molcel.2018.06.034 30057200

[B11] Li X. Zhang Z. Jiang H. Li Q. Wang R. Pan H. (2018b). Circular RNA circPVT1 promotes proliferation and invasion through sponging miR-125b and activating E2F2 signaling in non-small cell lung cancer. *Cell. Physiol. Biochem.* 51 2324–2340. 10.1159/000495876 30537738

[B12] Louis D. N. Perry A. Reifenberger G. von Deimling A. Figarella-Branger D. Cavenee W. K. (2016). The 2016 World Health Organization classification of tumors of the central nervous system: a summary. *Acta Neuropathol.* 131 803–820. 10.1007/s00401-016-1545-1 27157931

[B13] Lv X. Huang H. Feng H. Wei Z. (2020). Circ-MMP2 (circ-0039411) induced by FOXM1 promotes the proliferation and migration of lung adenocarcinoma cells in vitro and in vivo. *Cell Death Dis.* 11:426. 32513952 10.1038/s41419-020-2628-4 PMC7280516

[B14] Mangiola A. Anile C. Pompucci A. Capone G. Rigante L. De Bonis P. (2010). Glioblastoma therapy: going beyond Hercules Columns. *Expert Rev. Neurother.* 10 507–514. 10.1586/ern.09.158 20367204

[B15] Patop I. L. Kadener S. (2018). circRNAs in cancer. *Curr. Opin. Genet. Dev.* 48 121–127. 29245064 10.1016/j.gde.2017.11.007 PMC5877416

[B16] Ren S. Liu J. Feng Y. Li Z. He L. Li L. (2019). Knockdown of circDENND4C inhibits glycolysis, migration and invasion by up-regulating miR-200b/c in breast cancer under hypoxia. *J. Exp. Clin. Cancer Res.* 38:388. 31488193 10.1186/s13046-019-1398-2 PMC6727545

[B17] Song T. Xu A. Zhang Z. Gao F. Zhao L. Chen X. (2019). CircRNA hsa_circRNA_101996 increases cervical cancer proliferation and invasion through activating TPX2 expression by restraining miR-8075. *J. Cell. Physiol.* 234 14296–14305. 10.1002/jcp.28128 30633364 PMC13483104

[B18] Song X. Wang H. Wu J. Sun Y. (2020). Long noncoding RNA SOX2-OT knockdown inhibits proliferation and metastasis of prostate cancer cells through modulating the miR-452-5p/HMGB3 axis and inactivating Wnt/beta-catenin pathway. *Cancer Biother. Radiopharm.* 35 682–695. 10.1089/cbr.2019.3479 32407168

[B19] Song Y.F. Zhao L. Wang B. C. Sun J. J. Hu J. L. Zhu X. L. (2020). The circular RNA TLK1 exacerbates myocardial ischemia/reperfusion injury via targeting miR-214/RIPK1 through TNF signaling pathway. *Free Radic. Biol. Med.* 155 69–80. 10.1016/j.freeradbiomed.2020.05.013 32445866

[B20] Swiatek-Machado K. Kaminska B. (2020). STAT signaling in glioma cells. *Adv. Exp. Med. Biol.* 1202 203–222. 10.1007/978-3-030-30651-9_10 32034715

[B21] Tu Y. Zhong Y. Fu J. Cao Y. Fu G. Tian X. (2011). Activation of JAK/STAT signal pathway predicts poor prognosis of patients with gliomas. *Med. Oncol.* 28 15–23. 10.1007/s12032-010-9435-1 20135364

[B22] Van Meir E. G. Hadjipanayis C. G. Norden A. D. Shu H. K. Wen P. Y. Olson J. J. (2010). Exciting new advances in neuro-oncology: the avenue to a cure for malignant glioma. *CA Cancer J. Clin.* 60 166–193. 10.3322/caac.20069 20445000 PMC2888474

[B23] Wang H. He P. Pan H. Long J. Wang J. Li Z. (2018). Circular RNA circ-4099 is induced by TNF-alpha and regulates ECM synthesis by blocking miR-616-5p inhibition of Sox9 in intervertebral disc degeneration. *Exp. Mol. Med.* 50 1–14. 10.1038/s12276-018-0056-7 29651107 PMC5938034

[B24] Wu F. Han B. Wu S. Yang L. Leng S. Li M. (2019). Circular RNA TLK1 aggravates neuronal injury and neurological deficits after ischemic stroke via miR-335-3p/TIPARP. *J. Neurosci.* 39 7369–7393. 10.1523/jneurosci.0299-19.2019 31311824 PMC6759031

[B25] Wu L. Zhang M. Qi L. Zu X. Li Y. Liu L. (2019). ERalpha-mediated alterations in circ_0023642 and miR-490-5p signaling suppress bladder cancer invasion. *Cell Death Dis.* 10:635. 31455760 10.1038/s41419-019-1827-3 PMC6712013

[B26] Xiong J. Wang T. Tang H. Lv Z. Liang P. (2019). Circular RNA circMAN2B2 facilitates glioma progression by regulating the miR-1205/S100A8 axis. *J. Cell. Physiol.* 234 22996–23004. 10.1002/jcp.28860 31131447

[B27] Yan J. Wang Z. H. Yan Y. Luo H. N. Ren X. Y. Li N. (2020a). RP11156L14.1 regulates SSR1 expression by competitively binding to miR548ao3p in hypopharyngeal squamous cell carcinoma. *Oncol. Rep.* 44 2080–2092. 33000261 10.3892/or.2020.7762 PMC7551335

[B28] Yan J. Wei R. Li H. Dou Y. Wang J. (2020b). miR-452-5p and miR-215-5p expression levels in colorectal cancer tissues and their relationship with clinicopathological features. *Oncol. Lett.* 20 2955–2961. 10.3892/ol.2020.11845 32782612 PMC7400294

[B29] Yang W. Ju H. Y. Tian X. F. (2020). Circular RNA-ABCB10 suppresses hepatocellular carcinoma progression through upregulating NRP1/ABL2 via sponging miR-340-5p/miR-452-5p. *Eur. Rev. Med. Pharmacol. Sci.* 24 2347–2357. 32196586 10.26355/eurrev_202003_20501

[B30] Yang Y. Gao X. Zhang M. Yan S. Sun C. Xiao F. (2018). Novel role of FBXW7 circular RNA in repressing glioma tumorigenesis. *J. Natl. Cancer Inst.* 110 304–315. 10.1093/jnci/djx166 28903484 PMC6019044

[B31] Zhai W. Li S. Zhang J. Chen Y. Ma J. Kong W. (2018). Sunitinib-suppressed miR-452-5p facilitates renal cancer cell invasion and metastasis through modulating SMAD4/SMAD7 signals. *Mol. Cancer* 17:157. 30419914 10.1186/s12943-018-0906-x PMC6231268

[B32] Zhang P. Chen F. Z. Jia Q. B. Hu D. F. (2019). Upregulation of microRNA-133a and downregulation of connective tissue growth factor suppress cell proliferation, migration, and invasion in human glioma through the JAK/STAT signaling pathway. *IUBMB Life* 71 1857–1875. 10.1002/iub.2126 31381269

[B33] Zhang Z. Yang T. Xiao J. (2018). Circular RNAs: promising biomarkers for human diseases. *EBioMedicine* 34 267–274. 10.1016/j.ebiom.2018.07.036 30078734 PMC6116471

[B34] Zhu L. Wang C. Lin S. Zong L. (2020). CircKIAA0907 retards cell growth, cell cycle, and autophagy of gastric cancer in vitro and inhibits tumorigenesis in vivo via the miR-452-5p/KAT6B axis. *Med. Sci. Monit.* 26:e924160. 32722658 10.12659/MSM.924160 PMC7412918

